# “Association of MTHFR and MS/MTR gene polymorphisms with congenital heart defects in North Indian population (Jammu and Kashmir): a case–control study encompassing meta-analysis and trial sequential analysis”

**DOI:** 10.1186/s12887-022-03227-z

**Published:** 2022-04-25

**Authors:** Jyotdeep Kour Raina, Rakesh Kumar Panjaliya, Vikas Dogra, Sushil Sharma, Anupriya, Parvinder Kumar

**Affiliations:** 1 Institute of Human Genetics, University of Jammu Jammu and Kashmir, 180006 Jammu, India; 2 Department of Zoology, University of Jammu, Jammu and Kashmir, 180006 Jammu, India; 3 Department of Zoology Govt. Degree College, Samba, J&K Jammu, India; 4 Department of Neonatology, University Hospital Southampton, Hampshire, UK

**Keywords:** Polymorphism, MTHFR, Met-analysis, TSA

## Abstract

**Background:**

The risk of Congenital Heart Defects (CHD) is greatly influenced by variants within the genes involved in folate-homocysteine metabolism. Polymorphism in MTHFR (C677T and G1793A) and MS/MTR (A2756G) genes increases the risk of developing CHD risk, but results are controversial. Therefore, we conducted a case–control association pilot study followed by an up-dated meta-analysis with trial sequential analysis (TSA) to obtain more precise estimate of the associations of these two gene variants with the CHD risk.

**Methods:**

For case–control study, we enrolled 50 CHD patients and 100 unrelated healthy controls. Genotyping was done by PCR–RFLP method and meta-analysis was performed by MetaGenyo online Statistical Analysis System software. For meta-analysis total number of individuals was as follows: for *MTHFR* C677T 3450 CHD patients and 4447 controls whereas for *MS* A2756G 697 CHD patients and 777 controls.

**Results:**

Results of the original pilot study suggested lack of association for *MTHFR* C677T and *MS* A2756G polymorphism with risk of CHD whereas *MTHFR* G1793A was significantly associated with the disease. On performing meta-analysis, a significant association was observed with *MTHFR* C677T polymorphism but not with *MS* A2756G. Trial sequential Analysis also confirmed the sufficient sample size requirement for findings of meta-analysis.

**Conclusions:**

The results of the meta-analysis suggested a significant role of *MTHFR* in increased risk of CHD.

## Introduction

Congenital heart diseases or defects (CHD) which share a significant proportion in CVD burden arises due to incomplete development of heart during the first 6-weeks of gestation [1]. The origin of CHD is diverse which can be associated with a syndrome or be isolated (non-syndromic). It is hypothesized that susceptibility of cardiac defects increases with dual interaction of key gene(s)/SNP-environmental factors which perturb normal cardiac developmental process during embryonic life. The risk of CHD is greatly influenced by variants within the genes involved in folate-homocysteine metabolism [2–4]. Many studies have revealed that the risk of CHD in new-borns of females carrying mutations in genes involved in folate metabolism can be reduced by maternal periconceptional use of multivitamins or folic acid [5], however, the mechanism underlying this effect is still under investigation. Folate and vitamin B12 are known to influence homocysteine concentration. Folates taken in diet are usually polyglutamates which are converted to simpler forms, particularly monoglutamates, dihydrofolate, tetrahydrofolate and finally to methylated form of folate i.e. 5, 10-methylenetetrahydrofolate (5,10-MTHF) and 5-methyltetrahydrofolate (5-MTHF) by a specialised enzyme of the pathway. Homocysteine and folate metabolism is dependent on a couple of genes performing their specific role but two genes namely MTHFR and MS are considered critical genes for development of diseased cardiovascular phenotypes. A common mutation, C677T (rs1801133), in exon 4 of the MTHFR gene results in decreased enzyme activity and contributes to increased plasma homocysteine, particularly in individuals with low folate status. Rady and co-workers reported a novel polymorphic site of the *MTHFR* gene at nucleotide position 1793 G to A transition in exon 11 (rs2274976) which results an arginine-to-glutamine change at codon 594 and modifies enzyme activity [6]. The A2756G mutation (rs1805087) in MS gene alters re-methylation process and is also associated with increased homocysteine levels and risk of CHD. Most of the research in relation to folate-homocysteine metabolising pathway with the risk of CHD is based on parent-of-origin effect. There are very few studies focussing on embryonic variation in candidate genes of folate-homocysteine metabolising pathway in association with the development of structural congenital heart malformations during early pregnancy. Consistent with this view, we attempted to perform a case–control pilot study involving evaluation of two important genes: MTHFR (C677T and G1793A) and MS (A2756G) gene variations with risk of CHD in Jammu region of UT of J&K, India. Further, we also performed an updated meta-analysis with trial sequential analysis to investigate the association between MTHFR (C677T and G1793A) and MS (A2756G) polymorphisms and risk of CHD with increased statistical power.

## Methodology

### Study population and area

The present study was ethically approved by Institutional Ethical Committee, University of Jammu. The present study was carried out on 150 children, out of whom 50 children (0–12 years) were confirmed cases of CHD and 100 children (below 18 years) were unrelated healthy controls belonging to Jammu region of Union Territory of Jammu and Kashmir. The CHD cases were enrolled from In-patient Department of Paediatrics whereas controls were recruited from Out-patient Department of Paediatrics, Shri Maharaja Gulab Singh (SMGS) hospital, Jammu. Data and blood collection was done after having an informed written consent from attendant or guardian of the children. The diagnosis and classification of CHD was based on the clinical and the echocardiography findings. The inclusion/exclusion criteria were followed wherein patients with any form of CHD were included whereas patients with syndromes and neural tube defects were excluded. Controls admitted to hospital for minor ailments with no history of CHD or other major abnormality and also children visiting for blood typing were recruited for the study under reference. Power of the study for sample size calculation was done by using online tool based on mean and standard deviation of two groups of study subjects, two tail test and with alpha value of 5% (https://www.sphanalytics.com/statistical-power-calculator-using-average-values/). The power of the study obtained was more than 80%.

### Blood collection and DNA isolation

500 μl**-**1 ml of blood was collected in EDTA coated vacutainers from each child by trained paramedical staff of the Hospital. Isolation of DNA from whole blood was carried out using commercially available kits (DNeasy Blood and Tissue Kit, QIAGEN). The quantitative and qualitative analysis of isolated DNA was performed by spectrophotometry and 1.5% agarose gel electrophoresis respectively.

### Genotyping

**Fig. 1:**
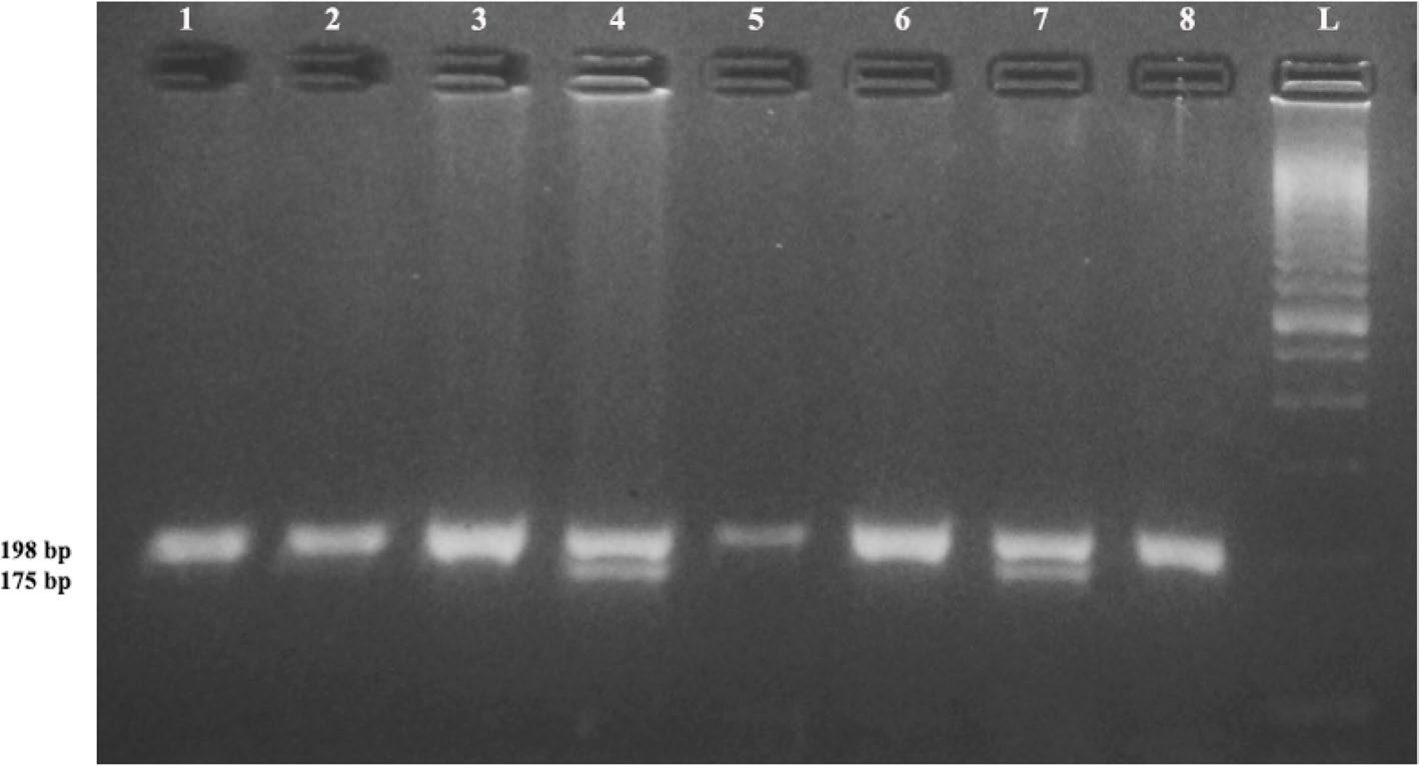
RFLP Gel Image of *MTHFR* C677T Polymorphism. L: 100 bp Ladder; Lane 1,2,3,5,6,8: 198 bp (CC: Wild)**;** Lane 4,7: 198 + 175 + 23 bp (CT: Hetero). *23 bp band will not be visible on agarose gel

**Fig. 2:**
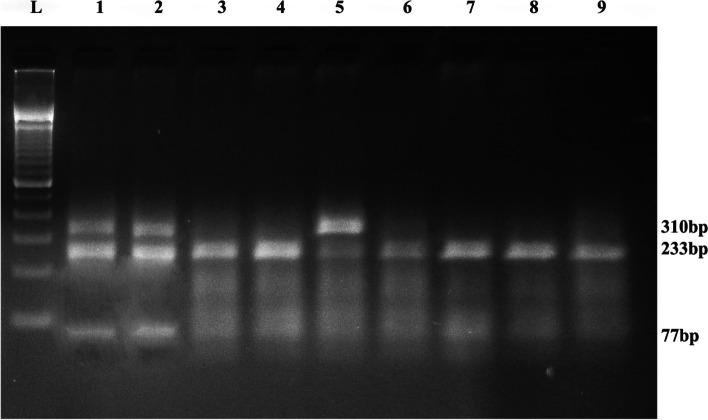
RFLP Gel Image of *MTHFR* G1793A Polymorphism. L: 100 bp Ladder; Lane 3,4,6,7,8: 233 + 77 bp (GG: Wild)**;** Lane 1,2,5: 310 + 233 + 77 bp (GA: Hetero)

**Fig. 3:**
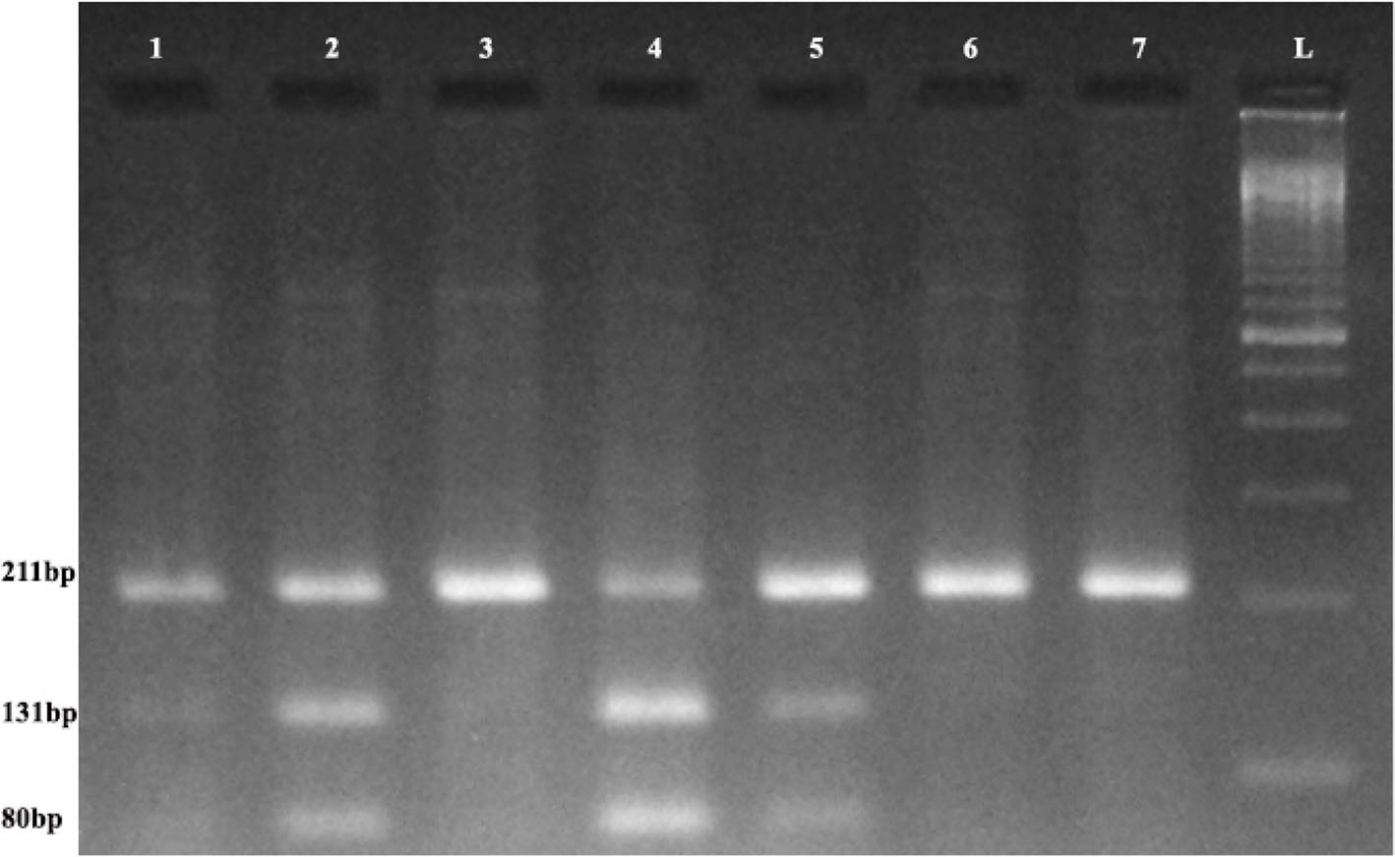
RFLP Gel Image of *MS* A2756G Polymorphism. L: 100 bp Ladder; Lane 1,2,6,7: 211 bp (AA: Wild)**;** Lane 2,4,5: 131 + 80 + 211 bp (AG: Hetero)

Genotyping was performed by polymerase chain reaction-restriction fragment length polymorphism (PCR–RFLP) technique. Briefly, PCR was carried out in a reaction volume of 25 μl each in thin walled tubes, consisting of 5.0 μl of PCR buffer (10X), 2.5 μl of MgCl2 (25 mM), 0.5 μl of dNTPs (10 mM), 0.5 μl (100 pmol/µl) of each of the forward and reverse primers, 0.3 μl (5unit/μl) of Taq DNA polymerase enzyme and 2 μl (40 ng) of genomic DNA. PCR amplification was carried out using the Veriti, Applied Biosystems by life technology, Singapore and amplification and RFLP conditions for all the three polymorphisms are given in Table 1. The gel images of PCR–RFLP for *MTHFR* (C677T and G1793A) and *MS* (A2756G) polymorphisms with band sizes have been depicted in Fig. 1, 2 and 3 respectively.

**Table 1:** Details of Primer sequence, amplification conditions and restriction enzymes

Gene polymorphism	Primer sequence	Amplicon (bp)	PCR conditions	Restriction enzymes	Genotypes	Reference
*MTHFR* C677T (rs1801133)	5’-TGA AGG AGA AGG TGT CTG CGG GA-3’ (F) 5’-AGG ACG GTG CGG TGA GAG TG-3’ (R)	198	Pre-Denaturation: 94 °C/ 2 min Denaturation: 94 °C/ 30 s Annealing: 62 °C/ 60 s Extension: 72 °C/ 30 s Final Extension: 72 °C/ 7 min. (40 cycles)	*HinfI*	CC = 198 bp CT = 198, 175 & 23 bp TT = 175 & 23 bp Figure 1	McBride et al., 2004 [36]
*MTHFR* G1793A (rs2274976)	5’-CTC TGT GTG TGT GTG CAT GTG TGC G-3’ (F) 5’-GGG ACA GGA GTG GCT CCA ACG CAG G-3’ (R)	310	Pre-Denaturation: 94 °C/ 1 min Denaturation: 94 °C/ 1 min Annealing: 67 °C/ 1 min Extension: 72 °C/ 1 min Final Extension: 72 °C/ 7 min. (40 cycles)	*BsrbI*	GG = 233 & 77 bp GA = 310, 233 & 77 bp AA = 310 bp Figure 2	Rady et al., 2002 [6]
*MS* A2756G (rs185087)	5’- TGT TCC AGA CAG TTA GAT GAA AAT C-3’ (F) 5’- GAT CCA AAG CCT TTT ACA CTC CTC-3’ (R)	211	Pre-Denaturation: 95 °C/ 4 min Denaturation: 95 °C/ 1 min Annealing: 61 °C/ 1.5 min Extension: 72 °C/ 1 min Final Extension: 72 °C/ 7 min. (35 cycles)	*HaeIII*	AA = 211 bp AG = 211, 131 & 80 bp GG = 131 & 80 bp Figure 3	Sahiner et al., 2014 [25]

### Statistical analyses

Genotypic frequency as well as allelic frequency was calculated by gene counting method. Hardy–Weinberg equilibrium (HWE) analysis and the differences in genotypic frequencies between two study groups were examined by using Pearson’s goodness of fit Chi-square test. To assess the association, odds ratios (OR) with 95% CI were calculated under different genetic models by using Statistical Package for Social Sciences (SPSS-version 20) software and also by another method provided by the Institute of Human Genetics accessed via the link: http://ihg.gsf.de/cgi-bin/hw/hwa1.pl. A *p*-value of < 0.05 was considered as statistically significant.

### Meta-analysis

#### Literature search

Research papers (published up to February, 2021) examining the association between *MTHFR* C677T, *MTHFR* G1793A and *MS* A2756G polymorphisms and congenital heart defects were extracted from databases such as PubMed, Science direct, Proquest, Ovid and Google Scholar. Key words used for the database search were as follows: methylenetetrahydrofolate reductase; *MTHFR* gene polymorphisms; Methionine synthase; *MS/MTR* gene polymorphisms; Congenital heart defects; Congenital heart diseases; *MTHFR* C677T; *MTHFR* G1793A and *MS/MTR* A2756G. Reference records of studies included in our meta-analysis were manually searched for possible eligible articles.

#### Inclusion and exclusion criteria

The inclusion/exclusion criteria used for screening of eligible study are given in Table 2.

**Table 2:** Inclusion/Exclusion criteria for eligible studies

Studies included	Studies excluded
● Studies with Case–control designs ● Report of the association between the *MTHFR* C677T, *MTHFR* G1793A and *MS* A2756G polymorphism and the risk of CHD ● Studies that included Pediatric participants ● Studies that follow Hardy Weinberg equilibrium (HWE) ● Studies with sufficient data ● Studies in English language	● Case reports ● Meta analysis and review articles ● Studies without control group ● Studies with abstract only ● Studies that include maternal/ paternal cases only ● Studies without detailed genotype data ● Studies that are associated with other diseases like CVD’s, thrombosis, coronary artery defects etc

#### Data extraction and quality assessment

From each eligible study, the following data were extracted by the two investigators independently using a standardized form: first author, publication year, country of origin, ethnicity, number of cases and controls, genotype frequency, source of controls, genotyping method, and Hardy–Weinberg equilibrium (HWE). We investigated the quality of each study based on the nine-point Newcastle–Ottawa Scale (NOS). The characteristics and results of NOS for all the included studies are shown in Table 3. The NOS scores for all eligible studies in this Meta analysis exceeded 6 points, indicating that our analysis is updated and is of good quality.

**Table 3:** Characteristics of the included studies in the meta-analysis

Study	Age Group/Mean age of cases	Mean age of controls	Diagnostic criteria	Source of controls	Country/ Region	Ethnicity	Genotyping Method	Cases				Controls				NOS	HWE
								**CC**	**CT**	**TT**	**Total**	**CC**	**CT**	**TT**	**Total**		
Junker et al*.,* 2001 [9]	0–16	Age matched	Echocardiography excluding DS or Chromosomal abnormality	HB	Germany	Caucasian	PCR–RFLP	51	42	21	114	129	78	21	228	9	0.0751
Lee et al*.,* 2005 [10]	Children	-	Confirmed CHD patients for cardiac catheterization	HB (cord blood from healthy foetuses)	Taiwan	Asian	DHPLC	110	89	14	213	114	68	13	195	9	0.5128
Li et al*.,* 2005 [11]	Children	Age matched	Registered patients of birth defects confirmed for CHD	HB	China	Asian	PCR–RFLP	30	95	58	183	22	57	24	103	9	0.2766
Shaw et al*.,* 2005 [12]	0–1 year & foetuses with CHD	Age matched	Conotruncal heart cases confirmed by Echocardiography	PB	America	Caucasian	DIRECT SEQUENCING	69	68	16	153	180	202	52	434	9	0.6836
Zhu et al*.,* 2006 [13]	6.2 yrs	8.4 yrs	Confirmed CHD by Echocardiography	PB	China	Asian	PCR–RFLP	3	7	12	22	22	57	24	103	9	0.2766
Zhu et al*.,* 2006 [13]	6.2 yrs	8.4 yrs	Confirmed CHD by Echocardiography	PB	China	Asian	PCR–RFLP	4	15	15	34	22	57	24	103	9	0.2766
van Beynum et al*.,* 2006 [4]	3.4 yrs	9.4 yrs	Echocardiography excluding NTD, cleft palate/lip, detected genetic abnormalities, known syndromes, and Vacterl- association	PB	Caucasian	Caucasian	PCR–RFLP	79	66	20	165	98	104	18	220	8	0.1842
Galdieri et al., 2007 [14]	0–11 yrs	-	Isolated cardiopathies (not associated with genetic syndromes or other malformations) confirmed by echocardiogram or cardiac catheterization	HB	Brazil	Caucasian	DIRECT SEQUENCING	30	21	7	58	18	14	6	38	9	0.2631
van Driel et al*.,* 2008 [15]	16.8 months	16.7 months	Confirmed CHD by echocardiography and/or cardiac catheterization and/or surgery	PB	European	Caucasian	Real time PCR, RFLP	99	103	27	229	119	107	25	251	9	0.8951
Xu et al*.,* 2010 [16]	6.50 yrs	6.69 yrs	Non-syndromic CHD cases confirmed by echocardiography	HB	China	Asian		162	244	96	502	151	261	115	527		0.9115
Kuehl et al*.,* 2010 [17]	Infants before one year of age	Age matched	Confirmed CHD by echocardiography and/or cardiac catheterization and/or surgery	PB	America	Caucasian	DIRECT SEQENCING	12	33	10	55	134	134	32	300	7	0.8611
Oberman-Borst et al*.,* 2011 [18]	17 months	17.3 months	Confirmed CHD by echocardiography and/or cardiac catheterization and/or surgery	PB	Netherlands	Caucasian	DIRECT SEQUENCING	64	66	9	139	92	76	15	183	8	0.9
Kotby et al*.,* 2012 [19]	31.5 months	32.7 months	Conotruncal heart defects excluding syndrome CHD	PB	Egypt	Caucasian	PCR–RFLP	12	14	4	30	20	8	2	30	8	0.3613
Gong et al*.,* 2012 [20]	2.27 yrs	1.58 yrs	Non-syndromic CHD cases confirmed by echocardiography and /or surgery	HB	Chinese Han population	Asian	MALDI-ToF–MS	45	123	76	244	43	72	21	136	8	0.3088
El-Abd et al*.,* 2012 [21]	Neonates	Neonates	Confirmed CHD except congenital heart disease associated with chromosomal anomalies and genetic syndromes, pre-mature infants (< 37 weeks gestation) and maternal diabetes, malabsorption, wasting syndromes, or any condition associated with folate deficiency	HB	Egypt	Caucasian	PCR–RFLP	7	12	7	26	13	5	0	18	9	0.4938
Wang et al*.,* 2013 [22]	-	-	Confirmed CHD by echocardiography	HB	China	Asian	SNaPShot genotyping, sequencing	59	76	25	160	53	100	35	188	9	0.3124
Kocakap et al*.,* 2014 [23]	3.7 yrs	8.7 yrs	Patients w ith echocardiographically proven conotruncal heart defect	HB	Turkey	Caucasian	HRM, PCR–RFLP, Sequencing	40	33	2	75	43	44	8	95	9	0.4841
Chao et al*.,* 2014 [24]	46.7 yrs	50.9yrs	Patients undergoing PDA ligation except patients diagnosed with diseases due to chromosomal defect or those born prematurely	HB	Taiwan	Asian	PCR–RFLP	10	5	2	17	19	12	3	34	8	0.5863
Mohamad et al*.,* 2014 [8]	Paediatric cases	> 21 years	Non-syndromic CHD patients confirmed by echocardiography	PB	Malaysians	Asian	PCR–RFLP	118	32	0	150	131	19	0	150	7	0.4076
Sahiner et al*.,* 2014 [25]	7.63 yrs	-	Non-syndromic CHD patients confirmed by echocardiography	HB	Turkey	Caucasian	PCR–RFLP	69	53	14	136	47	39	7	93	9	0.7791
Li et al*.,* 2015 [26]	-	-	Clinically confirmed CHD patients by echocardiography	HB	China	Asian	DIRECT SEQUENCING	31	78	41	150	59	66	25	150	9	0.3756
Shi et al*.,* 2015 [27]	-	-	Clinically confirmed CHD patients by echocardiography	PB	China	Asian	PCR–RFLP	55	68	30	153	70	101	45	216	8	0.4437
Wang et al*.,* 2016 [28]	1.46 yrs	3.08 yrs	Non-syndromic CHD patients confirmed by echocardiogram or cardiac catheterization	HB	Chinese Han population	Asian	Taq-Man allelic discrimination assay	14	73	60	147	49	84	35	168	9	0.9278
Noori et al*.,* 2017 [29]	4.2 yrs	4.9 yrs	confirmed CHD patients by echocardiography, cardiac catherization and surgical procedures	HB	Iran	Asian	Tetra-ARMS PCR	95	51	7	153	100	46	1	147	9	0.0781
Wang et al*.,* 2018 [30]	-	-	Conotruncal heart defects CHD patients by echocardiography	HB	China	Asian	DIRECT SEQUENCING	8	48	36	92	70	117	50	237	7	0.9316
Present study 2020	23.24 months	59.26 months	Non-syndromic CHD patients confirmed by echocardiography and surgical procedures	HB	Indian	Asian	PCR–RFLP	44	4	2	50	90	9	1	100	8	0.1796

#### Statistical analysis for meta-analysis

The association between the selected polymorphisms and congenital heart defects was evaluated for each study by the crude odds ratios (ORs) with 95% confidence intervals (CIs). For each study, HWE was assessed by the chi-square goodness of fit test. For all studies, we estimated the association under three different genetic models [Allele contrast, dominant model and recessive model]. Statistical heterogeneity between studies was assessed by Cochran’s Q test and I-square (I^2^) > 50% indicated the significance [31]. When I^2^ > 50%, a random-effect model should be taken otherwise fixed model is used. To calculate the OR and draw inference for each study, we used both random effects model and fixed effect model. Sensitivity analyses were conducted by omitting any single study, which predisposed the observed heterogeneity excessively and there should be no change in OR’s. Egger’s test and Begg’s funnel plot is used to solve the problem of Publication bias. All statistical analyses were performed in the MetaGenyo online Statistical Analysis System software [32].

#### Trial sequential analysis (TSA)

Meta-analysis may result in Type I error owing to an increased risk of random errors (play of chance) which can be due to dispersed data and repeated significance testing. Bias from low trial with low methodological qualities, publication bias and small trial bias may result in false *p*-value. Trial Sequential analysis is a methodology that can be used in meta-analysis to control random errors, and to assess whether the studies included in the meta-analysis have surpassed the requisite sample size. TSA was performed to calculate the required information size on the basis of overall 5% risk of Type-I error and a power of 80% for checking the reliability of meta analysis [33].

## Results

### Case–control study

Based on echocardiography reports, the different CHD phenotypes were categorised (Table 4). The observed prevalence of different CHD phenotypes in present study was highest for ventricular septal defect (VSD: 34%) and atrial septal defect (ASD: 26%) followed by tetralogy of fallot (TOF: 14%) and patent ductus arteriosus **(**PDA: 8%) and least for endocardial cushion defect (6%). The frequency of complex CHD forms (more than one CHD condition) were as follows: 4% for ASD with PDA, 2% for VSD with AV-canal defect, 4% for VSD with pulmonary arterial hypertension (VSD-PAH) and 2% for endocardial cushion defect along with dextrocardia.

**Table 4:** Prevalence of CHD phenotypes in present study

Type of CHD	No. of Cases (*N* = 50)	Percentage (%)
Ventricular septal defect (VSD)	17	34%
Atrial septal defect (ASD)	13	26%
Tetralogy of fallot (TOF)	7	26%
Patent ductus arteriosus **(**PDA)	4	8%
Endocardial cushion defect	3	6%
ASD with PDA	2	4%
VSD with peripheral arterial hypertension	2	4%
VSD with AV-canal defect	1	2%
Endocardial cushion defect along with dextrocardia	1	2%

The genotypic and allelic frequencies along with Chi square values for Hardy–Weinberg calculations for the all the three polymorphisms in study participants are depicted in Table 5. There observed frequencies of genotypes were in concordance with HWE in both the groups for all the polymorphisms except for MTHFR C677T in patient group. The genotypic frequency of CC, CT and TT (MTHFR C677T) in CHD patients was 88%, 8% and 4% whereas in controls it was 90%, 9% and 1% respectively. The frequency of variant allele T (0.08) was higher in CHD patients than controls (0.05) whereas wild allele C was reported to be in slightly higher frequency in controls (0.95) as compared to patients (0.92). The genotypic frequencies for MTHFR G1793A in CHD patients were 58%, 38% and 4% for GG, GA and AA respectively. The frequencies in control group were 90% for GG and 10% for GA genotypes; however we did not observe any AA genotype in controls. In general there was higher frequency of risk allele ‘A’ in CHD patients (0.23) in comparison to controls (0.05). The distribution of observed MS genotypes in CHD patients were 60%, 36% & 4% for AA, AG and GG genotypes respectively. In control group the distribution was as follows: 73% for AA, 26% for AG and 1% for GG genotype. The CHD patients were showing higher frequency of risk allele ‘G’ (0.22) than controls (0.14).

**Table 5:** Showing genotypic and allelic distribution of selected gene polymorphisms among cases and controls

Category	Genotypes/Alleles (%)					χ^2^	*p*-value
	***MTHFR* (C677T) polymorphism**						
	**CC (Wild)**	**CT (Hetero)**	**TT** **(Risk)**	**C** **(Wild)**	**T** **(Risk)**		
CHD Cases (*n* = 50)	44 (88%)	4 (8%)	2 (4%)	0.92	0.08	10.42	0.001*
Controls (*n* = 100)	90 (90%)	9 (9%)	1 (1%)	0.95	0.05	1.8	0.18
	***MTHFR* (G1793A) polymorphism**						
	**GG (Wild)**	**GA (Hetero)**	**AA (Risk)**	**G (Wild)**	**A (Risk)**		
CHD Cases (*n* = 50)	29 (58%)	19 (38%)	2 (4%)	0.77	0.23	0.27	0.61
Controls (*n* = 100)	90 (90%)	10 (10%)	0	0.95	0.05	0.28	0.60
	***MS* (A2756G) gene polymorphism**						
	**AA (Wild)**	**AG (Hetero)**	**GG (Risk)**	**A (Wild)**	**G (Risk)**		
CHD Cases (*n* = 50)	30 (60%)	18 (36%)	2 (4%)	0.78	0.22	0.12	0.73
Controls (*n* = 100)	73 (73%)	26 (26%)	1 (1%)	0.86	0.14	0.64	0.43

In order to investigate the possible association of these three polymorphisms with susceptibility of CHD, ORs with 95% confidentiality intervals was calculated for different genetic models which are presented in Table 6.

**Table 6:** Association between selected gene polymorphisms and CHD

MODEL	OR (95% CI)	*p*-value
***MTHFR* C677T polymorphism**		
Co-dominant		
CT vs CC	0.91 [0.27–3.12]	0.879
TT vs CC	4.09 [0.36–46.35]	0.22
Dominant		
CT + TT vs CC	1.23 [0.42–3.59]	0.71
Recessive		
TT vs CT + CC	4.12[0.36–46.63]	0.234
Allelic		
T vs C	1.49 [0.58–3.84]	0.40
***MTHFR* G1793A polymorphism**		
Co-dominant		
GA vs GG	**5.90 [2.46–14.11]**	**0.00002**^**b**^
AA vs GG	Not possible^a^	**-**
Dominant		
GA + AA vs GG	**6.52 [2.75–15.43]**	** < 0.0001**^**b**^
Recessive		
AA vs GA + GG	Not possible^a^	**-**
Allelic		
A vs G	**5.68 [2.58–12.48]**	** < 0.0001**^**b**^
***MS* A2756G polymorphism**		
Co-dominant		
AG vs AA	1.68 [0.81–3.52]	0.163
GG vs AA	4.87 [0.43–55.71]	0.20
Dominant		
AG + GG vs AA	1.80 [0.88–3.69]	0.11
Recessive		
GG vs AG + AA	4.12[0.36–46.63]	0.2
Allelic		
G vs A	1.73 [0.93–3.22]	0.08

^**a**^Some genotype combinations were not observed, so it was not possible to calculate odds ratio

^b^Significant values

For both *MTHFR* C677T and *MS* A2756G polymorphisms, we observed that even though the values calculated for ORs under different models were above 1, but none of the values reached statistical significance level (*p* > 0.05). The present study proclaimed lack of association of *MTHFR* C677T and *MS* A2756G gene polymorphism with the risk of CHD in our population. Furthermore, the GA vs GG genotype depicted a strong significant association of *MTHFR* G1793A gene polymorphism. The G vs A frequency showed that the allele ‘A’ is adding a significant risk of approximately 5.7 folds in the development of CHD in the studied population. Distribution of *MTHFR* haplotypes in cases & controls and their association towards CHD susceptibility is depicted in Table 7.

**Table 7:** Association of *MTHFR* haplotypes with risk of CHD

Variant *MTHFR* C677T/ G1793A	CHD Cases (*n* = 50)	Controls (*n* = 100)	OR (95% CI)	*p*-value^†^
C-A	0.230	0.050	**5.67 [2.58–12.48]**	**2.71e‐006**^**a**^
C-G	0.690	0.895	**0.26 [0.14–0.48]**	**1.00e‐005**^**a**^
T-G	0.080	0.055	1.49 [0.58–3.84]	0.40
T-A	0.000	0.000	-	-

^a^Significant values, ^†^Fisher’s *p*-value

The frequency of C-G haplotype was higher in both cases and controls (0.690 & 0.895 respectively). There was complete absence of T-A haplotype in both study groups. The haplotype combination C-A was significantly associated with CHD risk (OR = 5.67 [2.58–12.48], *p* = 2.71e‐006) and C-G was significantly involved in protection against CHD development (OR = 0.26 [0.14–0.48], *p* = 1.00e‐005) in the population under reference. By analysing LD scores in two study groups it was observed that the *MTHFR* variants were in complete LD in both patients (D' = 0.999, *r*^*2*^ = 0.026) and controls (D' = 1, *r*^*2*^ = 0.003).

### Meta-analysis

**Fig. 4:**
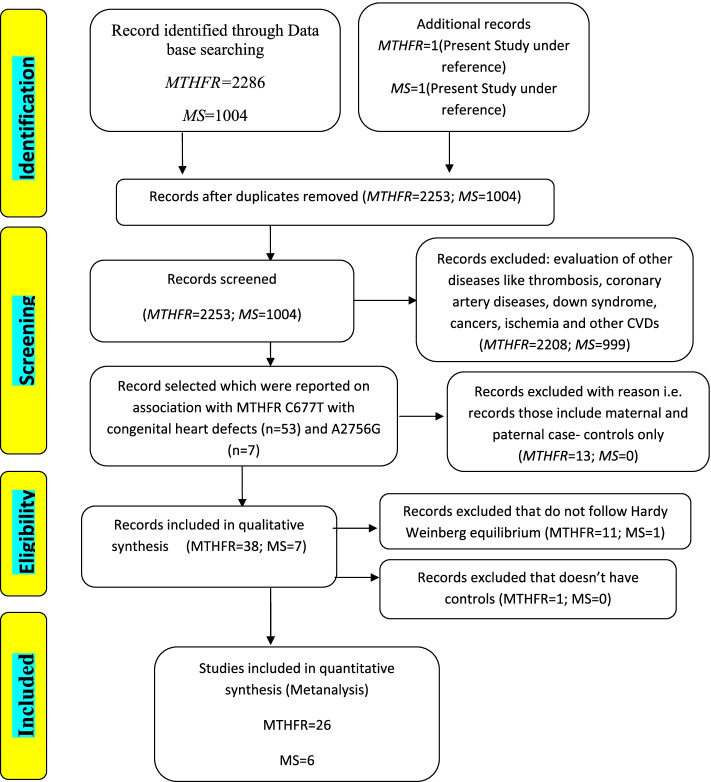
PRISMA flow diagram showing the selection of various studies for the meta –analysis

**Fig. 5:**
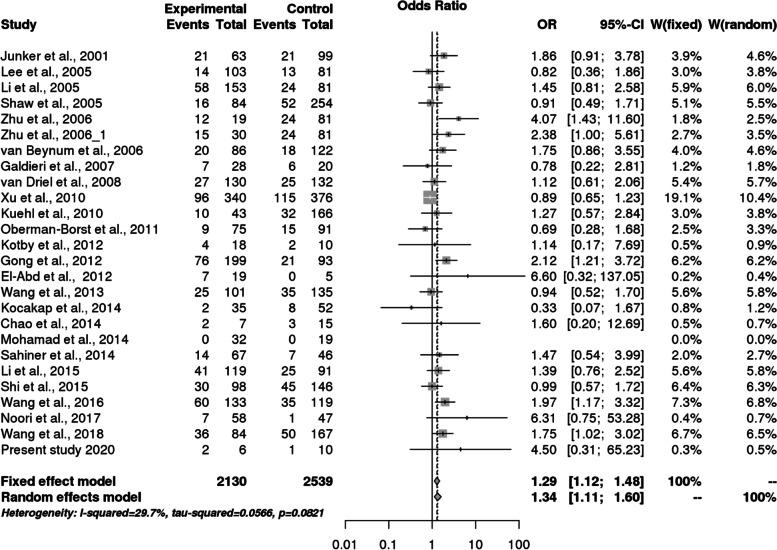
Pooled OR (Dominant model) and 95% CI for individual studies and pooled data for the association between the polymorphism C677T and congenital heart disease (CHD) in the overall population

**Fig. 6:**
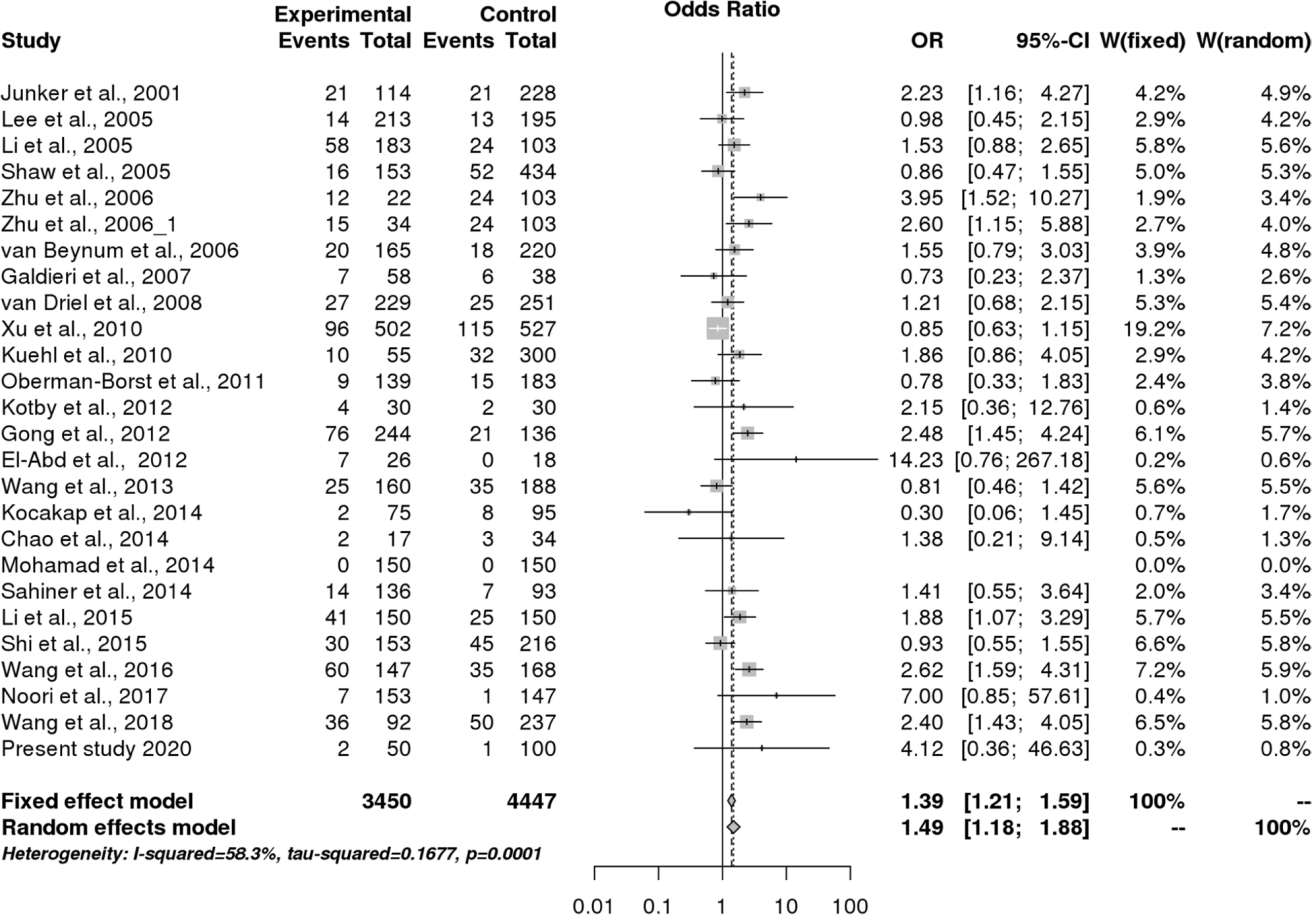
Pooled OR (Recessive model) and 95% CI for individual studies and pooled data for the association between the polymorphism C677T and congenital heart disease (CHD) in the overall population

We found 26 eligible studies having 3450 cases and 4447 controls with reference to *MTHFR* C677T polymorphism and 6 studies with 697 cases and 777 controls concerning *MS* A2756G polymorphism. The main study characteristics are summarized in Table 3. The study selection process has been depicted in PRISMA diagram (Fig. 4). By pooling all the studies, it was found that there is statistically significant association between *MTHFR* C677T polymorphism and congenital heart defects under all applied genetic models (Dominant model: OR = 1.38, 95% CI: 1.14- 1.69; recessive model: OR = 1.49, 95% CI: 1.83–1.87; allele model: OR = 1.33, 95% CI: 1.14–1.55) as shown in Table 8 and Fig. 5, 6, and 7. When we stratified the studies according to ethnicity, a significant association was observed between this locus and CHD only in Asian populations (Dominant model: OR = 1.50, 95% CI: 1.12- 2.01; recessive model: OR = 1.67, 95% CI: 1.21–2.31; allele model: OR = 1.42, 95% CI: 1.15- 1.76), but not in Caucasian populations (dominant model: OR = 1.24, 95% CI: 0.95- 1.62; Recessive model: OR = 1.27, 95% CI: 0.99–1.63; allele model: OR = 1.21, 95% CI: 0.97–1.50) as given in Table 8.

**Table 8:** Overall meta-analysis and subgroup analysis by ethnicity for *MTHFR* C677T polymorphism

Genetic Model	Number of studies	Test of association			Heterogeneity			Egger's test *p*- value
		**OR**	**95% CI**	***p*-value**	**Model**	***p*-value**	**I^2**	
**Overall**								
Allele contrast (T vs. C)	26	1.33	1.14–1.55	0.0002	Random	0.0001	0.7554	0.0259
Recessive model (TT vs. TC + CC)	25^a^	1.49	1.83–1.87	0.0007	Random	0.0001	0.5828	0.1945
Dominant model (TT + TC vs. CC)	26	1.38	1.14- 1.69	0.001	Random	0.0001	0.696	0.0068
Homozygous model (TT vs CC)	25^a^	1.75	1.26–2.44	0.001	Random	0.0001	0.7286	0.0699
Heterozygous model (TT vs CT)	25^a^	1.34	1.11–1.60	0.002	Random	0.02	0.5157	0.6033
**Caucasians**								
Allele contrast (T vs. C)	11	1.21	0.97–1.50	0.1	Random	0.0006	0.6755	0.1529
Recessive model (TT vs. TC + CC)	11	1.27	0.99–1.63	0.06	Fixed	0.1662	0.2933	0.8658
Dominant model (TT + TC vs. CC	11	1.24	0.95- 1.62	0.1	Random	0.003	0.6234	0.0657
Homozygous model (TT vs CC)	11	1.37	0.91- 2.07	0.1	Random	0.0237	0.5157	0.6033
Heterozygous model (TT vs CT)	11	1.78	0.91- 1.53	0.2	Fixed	0.5288	0	0.8349
**Asians**								
Allele contrast (T vs. C)	15	1.42	1.15- 1.76	0.001	Random	0.0001	0.7988	0.0765
Recessive model (TT vs. TC + CC)	14^a^	1.67	1.21–2.31	0.002	Random	0.0001	0.6958	0.1205
Dominant model (TT + TC vs. CC	15	1.50	1.12- 2.01	0.02	Random	0.0001	0.7438	0.0599
Homozygous model (TT vs CC)	14^a^	2.12	1.30–3.47	0.003	Random	0.0001	0.8067	0.08
Heterozygous model (TT vs CT)	14^a^	1.46	1.13–1.89	0.003	Random	0.03	0.4697	0.0834

^a^In one of the study, TT genotype is completely absent in one of the study group

**Fig. 7:**
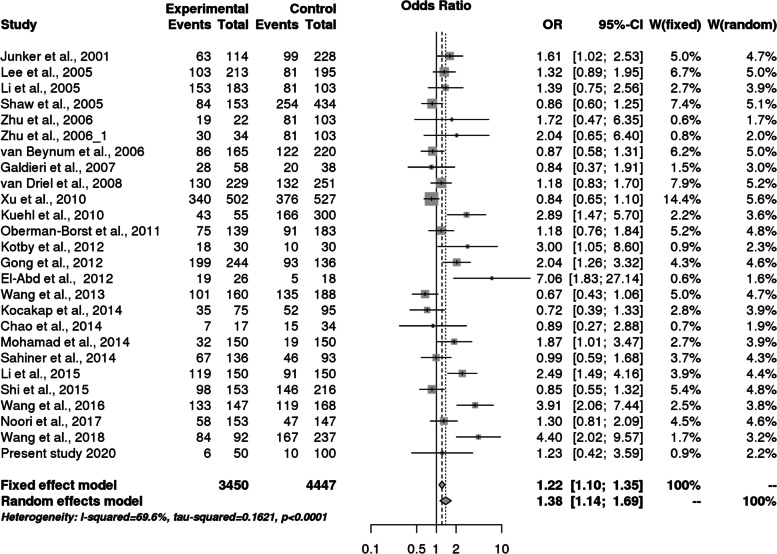
Pooled OR (Allele model) and 95% CI for individual studies and pooled data for the association between the polymorphism C677T and congenital heart disease (CHD) in the overall population

**Fig. 8:**
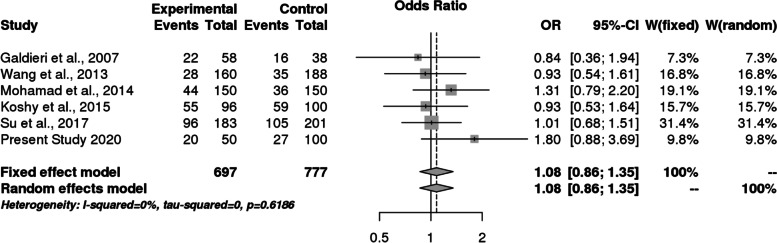
Pooled OR (Dominant model) and 95% CI for individual studies and pooled data for the association between the polymorphism MS/MTR A2756G and congenital heart disease (CHD) in the overall population

**Fig. 9:**
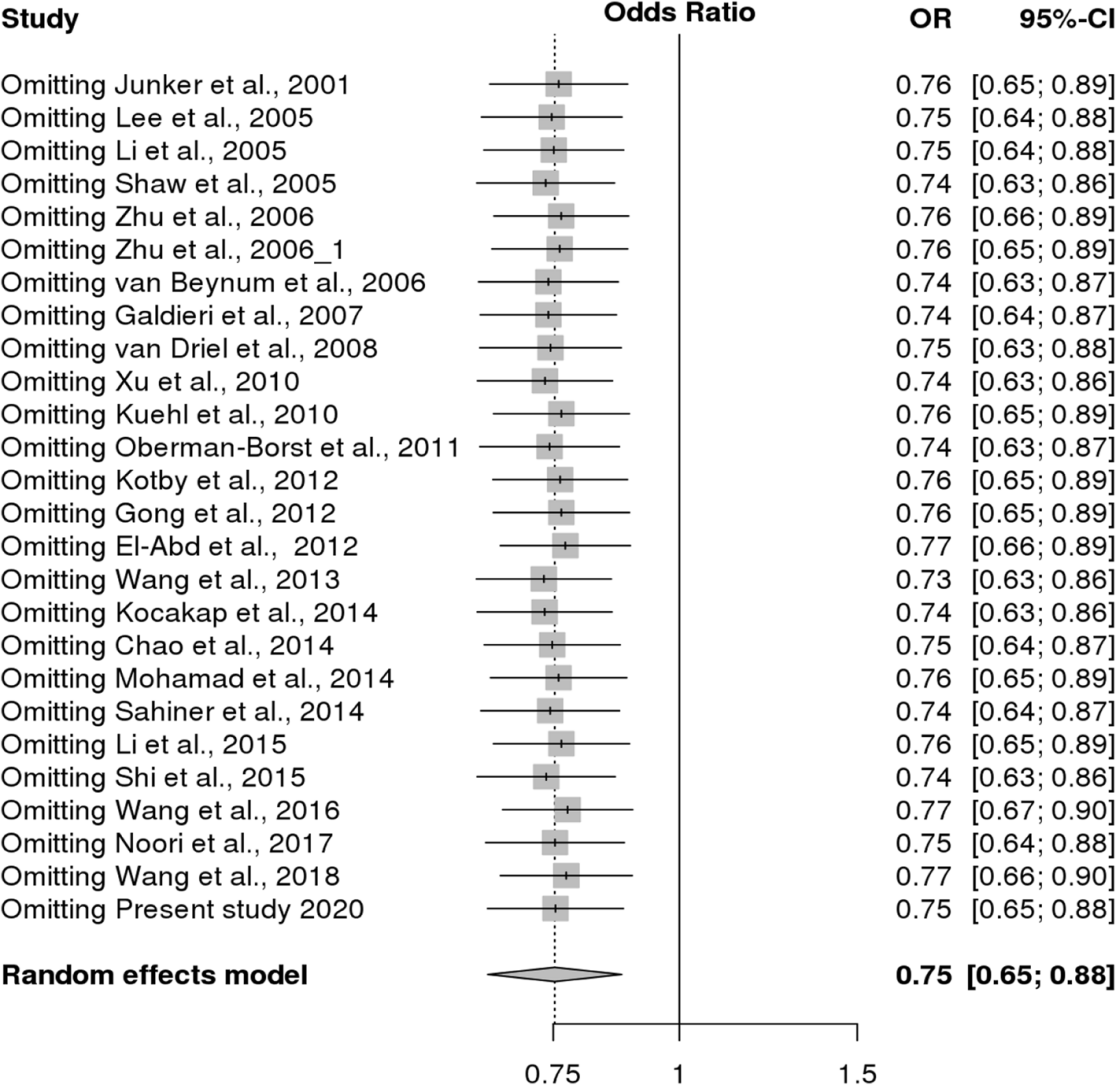
Sensitivity analysis of association between MTHFR C677T polymorphism and CHD

**Fig. 10:**
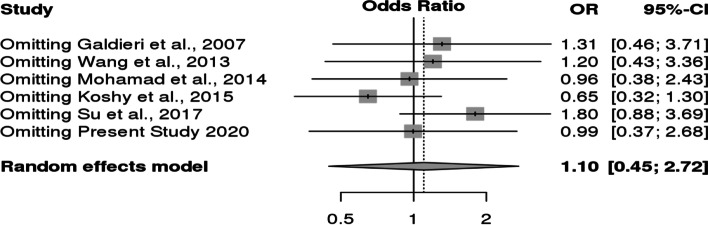
Sensitivity analysis of association between MS/MTR A2756G polymorphism and CHD

**Fig. 11:**
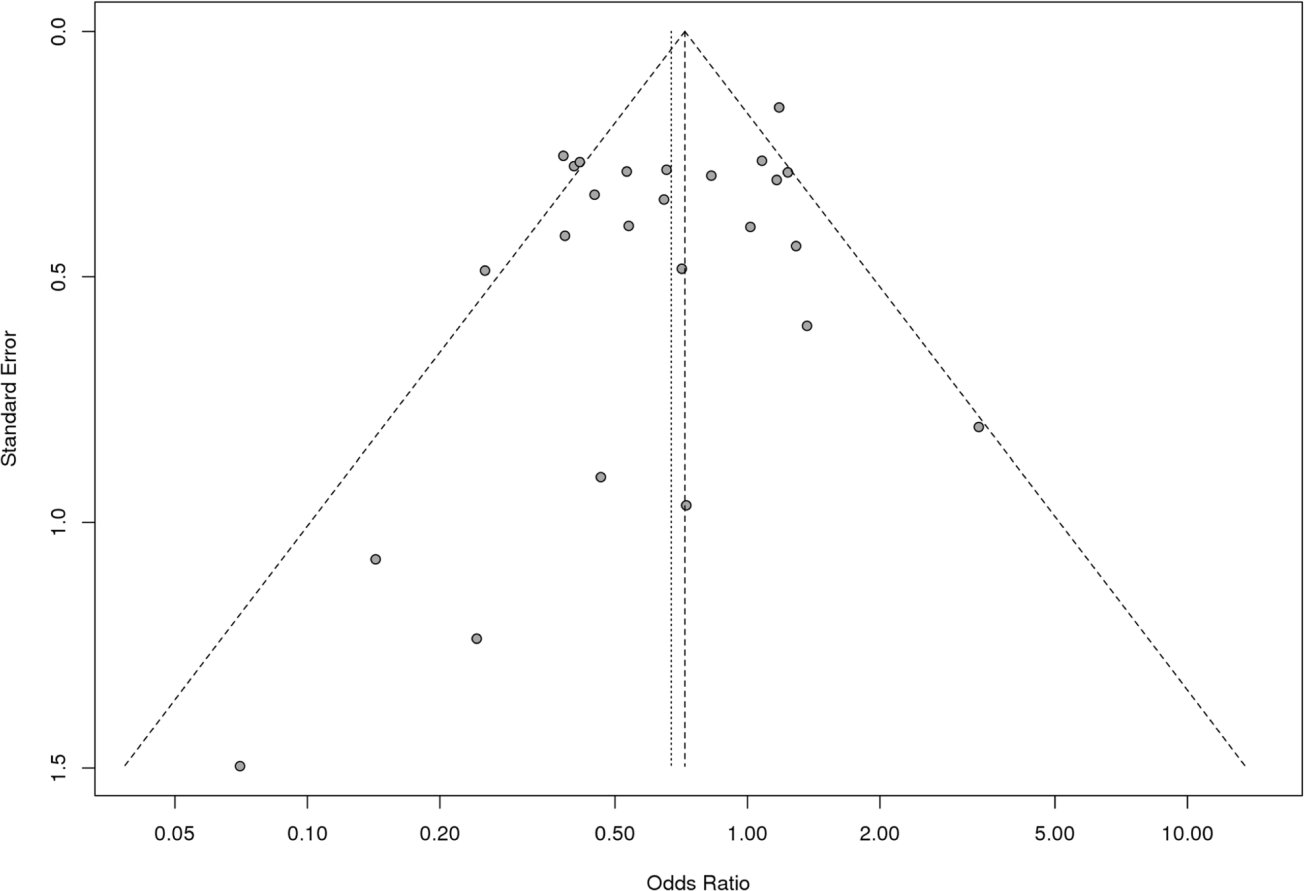
Funnel plot of the MTHFR C677T polymorphism and susceptibility to CHD (Dominant model) in the overall population

**Fig. 12:**
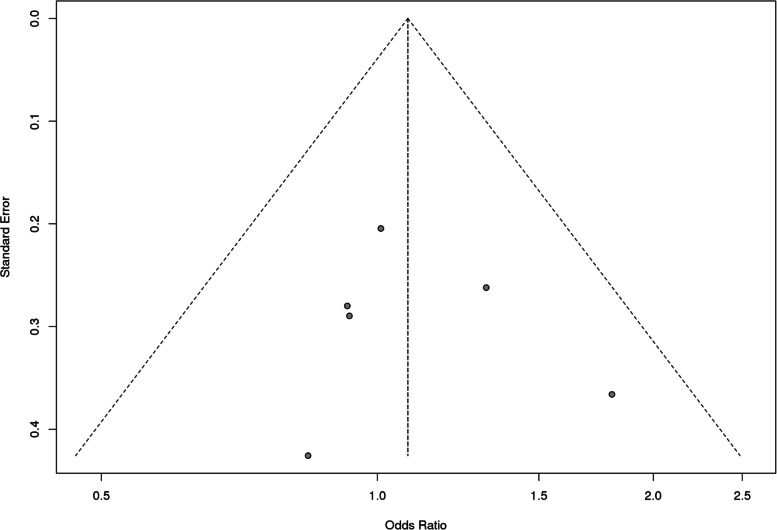
Funnel plot of the MS/MTR A2756G polymorphism and susceptibility to CHD (Dominant model) in the overall population

However, it was observed that Caucasian population was also showing association but it did not reach statistical significance. For *MS* polymorphism, none of the applied genetic models found association with CHD in overall population or even after subgrouping (Table 9 and Fig. 8). Sensitivity analysis for both *MTHFR* and *MS* revealed that there is no change in the pooled ORs by omitting individual studies (Fig. 9 and 10). The publication bias was also estimated by using funnel plot for log-odds ratio for dominant model against the reciprocal of its standard error (Fig. 11 and 12). Further Egger regression asymmetry test was also used to evaluate publication bias (Table 9). No publication bias was observed in the present meta-analysis. Meta- analysis could not be performed for MTHFR G1793A gene polymorphism as we were able to find only one study other than the study under reference. Meta-analysis could not be performed for MTHFR G1793A gene polymorphism as we were able to find only one study other than the study under reference.

**Table 9:** Overall meta-analysis and subgroup analysis by ethnicity for *MS* A2756G polymorphism

Genetic Model	Number of studies	Test of association			Heterogeneity				Egger's test *p*- value
		**OR**	**95% CI**	***p*-value**	**Model**	***p*-value**		**I^2**	
**Overall**									
Allele contrast (G vs. A)	6	1.05	0.88–1.26	0.6	Fixed	0.3		0.1993	0.4631
Recessive model (GG vs. AG + AA)	6	1.11	0.47–2.64	0.8	Random	0.07		0.5136	0.5171
Dominant model (GG + AG vs. AA)	6	1.08	0.86–1.35	0.5	Fixed	0.6		0	0.7422
Homozygous model (GG vs AA)	6	0.95	0.57–1.56	0.8	Fixed	0.1		0.4122	0.4344
Heterozygous model (GG vs AG)	6	1.10	0.45–2.72	0.8	Random	0.06		0.5204	0.5685
**Caucasians**									
Allele contrast (G vs. A)	3	0.95	0.75–1.19	0.6	Fixed	0.5		0	0.9501
Recessive model (GG vs. AG + AA)	3	0.86	0.30–2.47	0.8	Random	0.03		0.7067	0.9516
Dominant model (GG + AG vs. AA)	3	0.96	0.71–1.31`	0.8	Fixed	0.92		0	0.0379
Homozygous model (GG vs AA)	3	0.84	0.34–2.06	0.7	Random	0.1		0.5605	0.9324
Heterozygous model (GG vs AG)	3	0.87	0.26–2.91	0.82	Random	0.02		0.7476	0.9915
**Asians**									
Allele contrast (G vs. A)	3	1.25	0.93–1.69	0.1	Fixed	0.3	0.2455		0.6974
Recessive model (GG vs. AG + AA)	3	2.26	0.51–9.94	0.3	Fixed	0.4	0.0104		0.5599
Dominant model (GG + AG vs. AA)	3	1.24	0.89–1.73	0.21	Fixed	0.3	0.0785		0.5501
Homozygous model (GG vs AA)	3	2.42	0.55–10.69	0.2	Fixed	0.3	0.1005		0.577
Heterozygous model (GG vs AG)	3	1.95	0.43–8.78	0.4	Fixed	0.5	0		0.4763

### Trial Sequential Analysis (TSA)

**Fig. 13:**
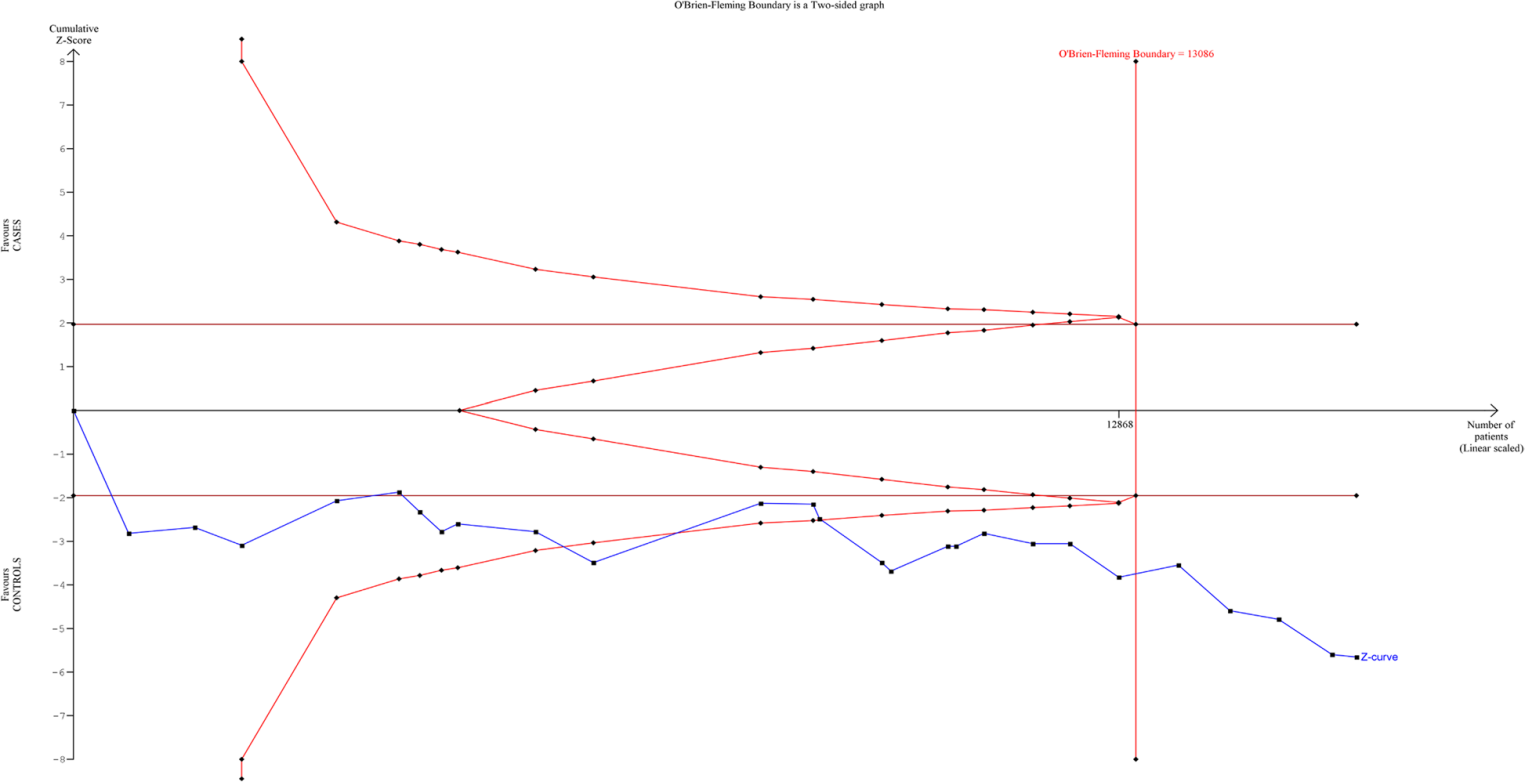
Trial Sequential Analysis (TSA) of the studies included in the meta analysis of MTHFR C677T gene polymorphism with CHD

Trial sequential analysis was performed to calculate the requisite sample size for the meta-analysis of MTHFR C677T gene polymorphism. It revealed that sufficient number of studies have been included in the meta-analysis of this polymorphism. The results of TSA were in accordance with the findings of the conventional meta-analysis and revealed that C677T polymorphism was significantly associated with the risk of CHD (Fig. 13). For *MS* A2756G polymorphism, TSA could not be performed owing to very little information of sample size which revealed that there is need of more replicas of case control studies to reach the conclusive remarks on role of said polymorphism in conferring risk of CHD. Similarly for MTHFR G1793A gene polymorphism, TSA could not be performed as only two studies were available for meta-analysis.

## Discussion

The folate-homocysteine metabolic pathway performs a paramount role in neural tube formation and cardiac development during embryogenesis. Low folate and high homocysteine levels are a closely related with the manifestation of congenital heart defects, which indicates that single nucleotide polymorphisms (SNPs) in the genes controlling this pathway may be the genetic risk factors for these disorders [34]. Therefore, we performed a case–control association study and an updated meta-analysis along with TSA to investigate the association of *MTHFR* and *MS* gene polymorphisms with risk of CHD. We did not find a significant association of MTHFR C677T and MS A2756G polymorphism with risk of CHD in our studied population. The results were consistent with studies done by various workers [5, 7, 35–38]. Regarding MTHFR G1793A polymorphism in link with CHD risk we found significant association under co-dominant, dominant and allelic model in present study. The genotypic frequencies reported in the present study were almost compatible with frequencies as reported by Toganel and co-workers and the investigators also observed a strong significant association this SNP with susceptibility of CHD [AA + GA vs GG: OR = 4.18; 95% CI (1.25- 13.98), *p* = 0.02] in a Romanian population whereas antithetical findings were reported in Chinese population [39, 40]. Xu and co-workers found that the variant genotypes of *MTHFR* G1793A polymorphism were significantly associated with a decreased risk of CHD, especially in patients with isolated peri-membranous VSD [40]. The correlation between the MTHFR G1793A gene polymorphism and the CHD risk has not been extensively studied so far. To the best of our knowledge there is no previous report from India and we are the first to analyse G1793A variation of MTHFR gene from North India. The present study is first of its kind concentrating on the effect of MTHFR (C677T and G1793A) haplotypes with vulnerability of CHD. The haplotype C-A was conferring nearly 5.7-fold disease risk and C-G haplotype was giving a shielding outcome of approximately 3.8-fold (1/0.26). Based on measure of LD, the two MTHFR SNPs were in complete LD in both CHD cases and controls. The possible limitations of the present study may be the enrolment of study samples from single region of UT J&K and lack of homocysteine measurements in the study subjects. Besides these limitations and to the best of our knowledge, the study under reference here is the first attempt that evaluates the association of MTHFR and MS gene polymorphisms in CHD.

Genetic association studies have been a powerful approach for identifying susceptibility genes for common diseases but it has been experienced that most of the initial positive associations were not reproduced in the subsequent replication studies because of small sample size or false-positive reports [41, 42]. Meta-analysis solves this problem as it increases the statistical power to detect gene–disease associations by combining results from the original and subsequent replication studies [42]. Similarly, when we conducted case–control association, we did not observe significant association of *MTHFR* C677T with risk of CHD, as it was a pilot study and carried on limited number of samples. But after performing meta-analysis, the results suggested a positive association of *MTHFR* C677T with the risk of CHD. The results of the overall analysis depicted an increased risk of CHD with the presence of *MTHFR* 677 T- allele in fetus. The putative risk allele-677 T had a 1.33 folds increased risk of CHD against the C-allele. From the subgroup analysis, the increased risk of the T-allele was widely detected in Asians but not in Caucasians. Our results are compatible with the previous Meta analyses that investigated the association of the MTHFR C677T polymorphism in CHD [34, 43]. Further, this association revealed through conventional meta-analysis has also been confirmed by performing Trial Sequential Analysis. Lack of association was reported for MS A2756G both in pooled and in sub-grouped meta-analysis and the findings are consistent with study done by Cai and co-workers [44]. The findings of MS polymorphism needs to be further investigated as there are not enough studies on association of this polymorphism with risk of CHD and during our search we also found only six eligible studies and TSA has not been performed in lieu of lack of sufficient number of studies. Further, we were not able to perform meta- analysis for MTHFR G1793A polymorphism as to best of our efforts; we found only a few case–control studies which were not sufficient for performing meta-analysis.

## Conclusion

In conclusion, the results of meta-analysis and TSA support the role of MTHFR C677T gene polymorphism as susceptibility factor for Congenital Heart Defects. For MTHFR G1793A and MS A2756G gene polymorphisms, there is need to perform large number of homogenous studies to evaluate these crude results further.

## Data Availability

The data and the material used in the research work under reference can be made available upon reasonable request from corresponding author.

## Abbreviations

CHD: Congenital Heart Defects
MTHFR: Methylenetetrahydrofolate reductase
MTR: 5-Methyltetrahydrofolate-Homocysteine Methytransferase
TSA: Trial sequential analysis
PCR: Polymerase Chain Reaction
RFLP: Restriction Fragment Length Polymorphism
PCR–RFLP: Polymerase Chain Reaction- Restriction Fragment Length Polymorphism
CVD: Cardiovascular Disease
5, 10-MTHF: 5, 10-Methylenetetrahydrofolate
5-MTHF: 5-Methyltetrafolate
MS: Methionine synthase
HWE: Hardy–Weinberg equilibrium
OR: Odd Ratio
CI: Confidence Interval
SPSS: Statistical Package for Social Sciences
NOS: Newcastle–Ottawa Scale
I^2^: I-square
*p*-value: Probability value
VSD: Ventricular septal defect
ASD: Atrial septal defect
TOF: Tetralogy of fallot
PDA: Patent ductus arteriosus
VSD-PAH: Ventricular septal defect with pulmonary arterial hypertension
LD: Linkage Disequilibrium
PRISMA: Preferred Reporting Items for Systematic Reviews and Meta-Analyses
SNPs: Single nucleotide polymorphisms
AV canal defect: Atrioventricular canal defect
